# Next generation single-domain antibodies against respiratory zoonotic RNA viruses

**DOI:** 10.3389/fmolb.2024.1389548

**Published:** 2024-05-09

**Authors:** Iris C. Swart, Willem Van Gelder, Cornelis A. M. De Haan, Berend-Jan Bosch, Sabrina Oliveira

**Affiliations:** ^1^ Cell Biology, Neurobiology and Biophysics, Department of Biology, Faculty of Science, Utrecht University, Utrecht, Netherlands; ^2^ Virology Section, Infectious Diseases and Immunology Division, Department Biomolecular Health Sciences, Faculty Veterinary Medicine, Utrecht University, Utrecht, Netherlands; ^3^ Pharmaceutics, Department of Pharmaceutical Sciences, Faculty of Science, Utrecht University, Utrecht, Netherlands

**Keywords:** single-domain antibodies, nanobodies, VHHs, respiratory zoonotic RNA viruses, influenza virus, coronavirus, sdAb bioengineering

## Abstract

The global impact of zoonotic viral outbreaks underscores the pressing need for innovative antiviral strategies, particularly against respiratory zoonotic RNA viruses. These viruses possess a high potential to trigger future epidemics and pandemics due to their high mutation rate, broad host range and efficient spread through airborne transmission. Recent pandemics caused by coronaviruses and influenza A viruses underscore the importance of developing targeted antiviral strategies. Single-domain antibodies (sdAbs), originating from camelids, also known as nanobodies or VHHs (Variable Heavy domain of Heavy chain antibodies), have emerged as promising tools to combat current and impending zoonotic viral threats. Their unique structure, coupled with attributes like robustness, compact size, and cost-effectiveness, positions them as strong alternatives to traditional monoclonal antibodies. This review describes the pivotal role of sdAbs in combating respiratory zoonotic viruses, with a primary focus on enhancing sdAb antiviral potency through optimization techniques and diverse administration strategies. We discuss both the promises and challenges within this dynamically growing field.

## 1 Introduction

The majority of recent viral outbreaks originate from the animal world through transmission of so-called zoonotic viruses (Jones et al., 2008). Currently, the most imminent pandemic threat comes from zoonotic RNA viruses belonging to the Coronaviridae and Orthomyxoviridae families, which cause respiratory infections in humans. Recently, three animal coronaviruses (CoVs) have emerged in humans causing severe disease outcomes: severe acute respiratory syndrome coronavirus 1 (SARS-CoV-1), Middle East respiratory syndrome-virus (MERS-CoV) and severe acute respiratory syndrome coronavirus 2 (SARS-CoV-2) which gave rise to the recent COVID-19 pandemic (Zhong et al., 2003; Zaki et al., 2012; Munir et al., 2020). In addition, influenza A and B viruses (IAV and IBV) cause annual epidemics, with IAV occasionally causing pandemics, as exemplified by the 2009 H1N1 pandemic (H1N1pdm09) flu outbreak (Smith et al., 2009; Flerlage et al., 2021). The inherent zoonotic potential and pandemic risk of these RNA viruses can be attributed, in part, to their high mutation rates caused by low fidelity RNA genome replication and fast viral evolution facilitated by recombination and reassortment events. Such genomic alterations may facilitate viral adaptation, enabling the viruses to persist in the population and enhancing their ability to cross species barriers (Alvarez-Munoz et al., 2021). These attributes emphasize the pressing need for effective antiviral strategies, with a broad reactivity of these antivirals being paramount to ensure activity against newly emerging viruses.

Currently, the landscape of antiviral approaches involves a combination of prophylactic and therapeutic measures. Vaccination plays a pivotal role in preventing viral infections, particularly within vulnerable segments of the population. Additional antiviral drugs aid in treating infectious diseases by mitigating symptom severity. However, efficacy of antiviral drugs and/or vaccines is challenged by the emergence of drug-resistant viral strains and limited effectiveness against newly emerging viruses. The dynamic nature of zoonotic viruses necessitates continuous innovation in the antiviral repertoire to effectively counteract their evolving strategies for host adaptation and immune evasion.

SdAbs constitute the variable domain of heavy-chain-only antibodies found in, amongst others, camelids such as llamas, dromedaries and camels (Arbabi Ghahroudi et al., 1997). Their distinctive and versatile characteristics distinguish them from conventional antibodies. Characterized by their small size and robustness, sdAbs can facilitate excellent tissue penetration and are suitable for administration via inhalation. Additionally, sdAbs can target various stages in the viral life cycle and can be easily bioengineered into optimized formats (De Vlieger et al., 2018). These attributes make sdAbs highly valuable for engineering innovative biotherapeutics with potent and broad antiviral activity against viral pathogens.

This review presents the latest advancements in optimizing camelid-derived sdAbs for combating respiratory zoonotic RNA virus infections. It explores cutting-edge bioengineering techniques aimed to enhance sdAb therapeutic potential, while discussing the associated challenges and promises. This review provides valuable insights for the development and administration of novel and potent sdAb-based antiviral interventions.

## 2 Single-domain antibodies

**FIGURE 1:**
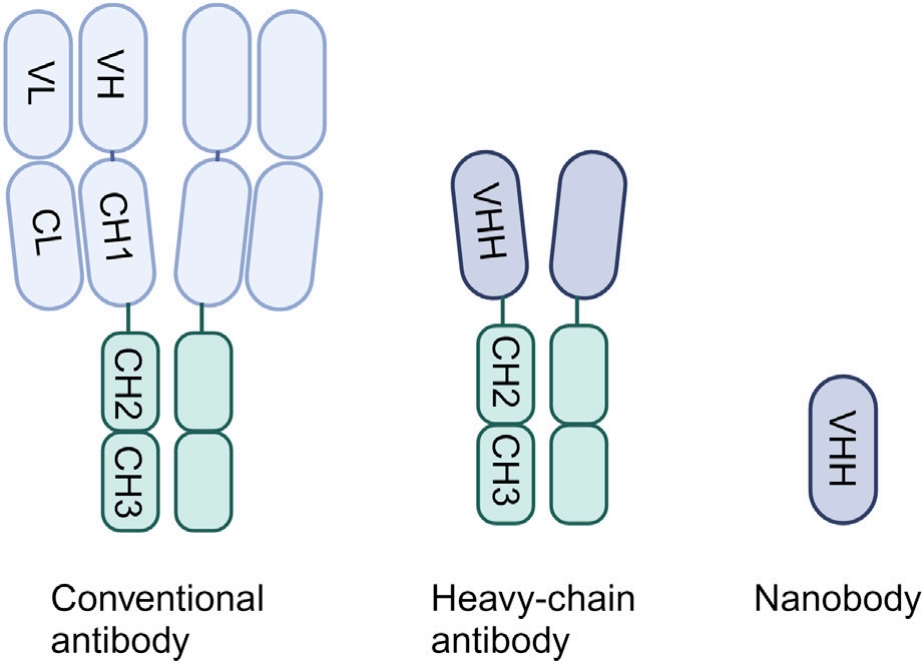
The structure of a conventional antibody, heavy-chain only antibody and single-domain antibody.

In 1993, it was discovered that a distinct class of antibodies could be found in the bloodstream of camelids, alongside the conventional antibodies, identified as heavy-chain antibodies (HCAbs) (Hamers-Casterman et al., 1993). Only 2 years later it was found that the immune system of cartilaginous fish, like sharks, also contains natural antibody isotypes composed of heavy chains only (Greenberg et al., 1995). Both conventional antibodies and HCAbs include the fragment crystallizable (Fc) tail. However, unlike typical antibodies, which consist of heavy chain and light chain heterodimers, HCAbs exhibit a singular structure comprising only two heavy chains in the form of homodimers, lacking accompanying light chains (Figure 1) (Padlan, 1994). Conventional antibodies bind the antigen through the variable domains of the heavy chain (VH) and light chain (VL), whereas for HCAbs binding is achieved by just a single domain: the variable domain of the heavy chain antibody (VHH). These single-domain antigen-binding fragments can be obtained from HCAbs and can be expressed independently (Arbabi Ghahroudi et al., 1997). They can be recognized by different names, namely, sdAbs, VHHs or nanobodies. Remarkably small, around 15 kDa, they display exceptional characteristics comparable to or even surpassing those of conventional antibodies.

SdAbs consist of three Complementarity-Determining Regions (CDR), with an unusually elongated CDR3 loop enabling effective targeting of concealed epitopes often inaccessible to conventional antibodies (Vu et al., 1997). This elongated CDR3 region also plays a pivotal role in intrinsic sdAb stability, showcasing exceptional robustness with high thermo- and chemo-stability (Kunz et al., 2018). Additionally, sdAbs present a hydrophilic surface, resulting in high solubility (Jin et al., 2023). Their single-domain nature and small size facilitates easy modification through bioengineering, commonly achieved by linking sdAbs into multivalent or multi-specific constructs. This multimerization can lead to an increase in binding affinity through avidity effects, potently enhancing sdAb neutralization potency or expanding the breadth of the sdAbs (Saerens et al., 2008; De Vlieger et al., 2018; Chi et al., 2022b). Moreover, multimerization of sdAbs targeting different epitopes may increase resilience against viral escape (Koenig et al., 2021; Walter et al., 2022). Furthermore, sdAbs can be fused with other moieties to extend their half-life, facilitate immune cell recruitment or facilitate efficient drug delivery (Saerens et al., 2008). Considering clinical application, sdAbs exhibit low immunogenicity in humans due to the high sequence homology to human variable VH domains, especially those derived from the VH3 gene family (Klarenbeek et al., 2015). This characteristic can be further enhanced through humanization strategies (Vincke et al., 2009). Additionally, their high stability enable nebulization, presenting a distinct advantage in various clinical scenarios (Gai et al., 2021).

The typical method for obtaining sdAbs involves immunizing camelids with antigens of interest. After which, HCAb mRNA is collected, and sdAb DNA sequences are subsequently cloned into surface display vectors. The constructed library allows the selection of sdAbs binding the antigen of interest through diverse methods, including phage, ribosome, or yeast display (Muyldermans, 2021). Once selected, these sdAbs can be cloned into expression vectors, allowing for their high-yield expression in various low-cost production systems, with bacterial production being the most commonly used (Liu and Huang, 2018). In addition to deriving sdAbs from camelids, they can also be sourced from transgenic mice that produce fully human HCAbs (Janssens et al., 2006; Drabek et al., 2022). This approach using transgenic mice omits the need for humanization, which is preferred when utilizing camelid-derived sdAbs. Furthermore, it demands lower quantities of antigen for immunization. However, due to the inherent hydrophobic interaction of these mice derived VH with free available VL domains, the VH domains exhibit higher instability and greater tendency to aggregate compared to the camelid derived sdAbs. Additionally, the advantageous elongated CDR3 region is exclusive to camelid derived sdAbs (Drabek et al., 2016; Bannas et al., 2017). As an alternative to deriving sdAbs from immune libraries, they can be selected from naïve or synthetic sdAb libraries (Zhao et al., 2022). The latter is increasingly preferred as it avoids the use of animals and can be employed for multiple targets due to the library’s non-specific nature. However, a drawback is the typically lower binding affinity of sdAbs selected from synthetic libraries, which can be overcome by *in vitro* affinity maturation to enhance their binding affinity (Liu and Yang, 2022; Valdés-Tresanco et al., 2022).

## 3 SdAbs targeting respiratory zoonotic viruses

**FIGURE 2:**
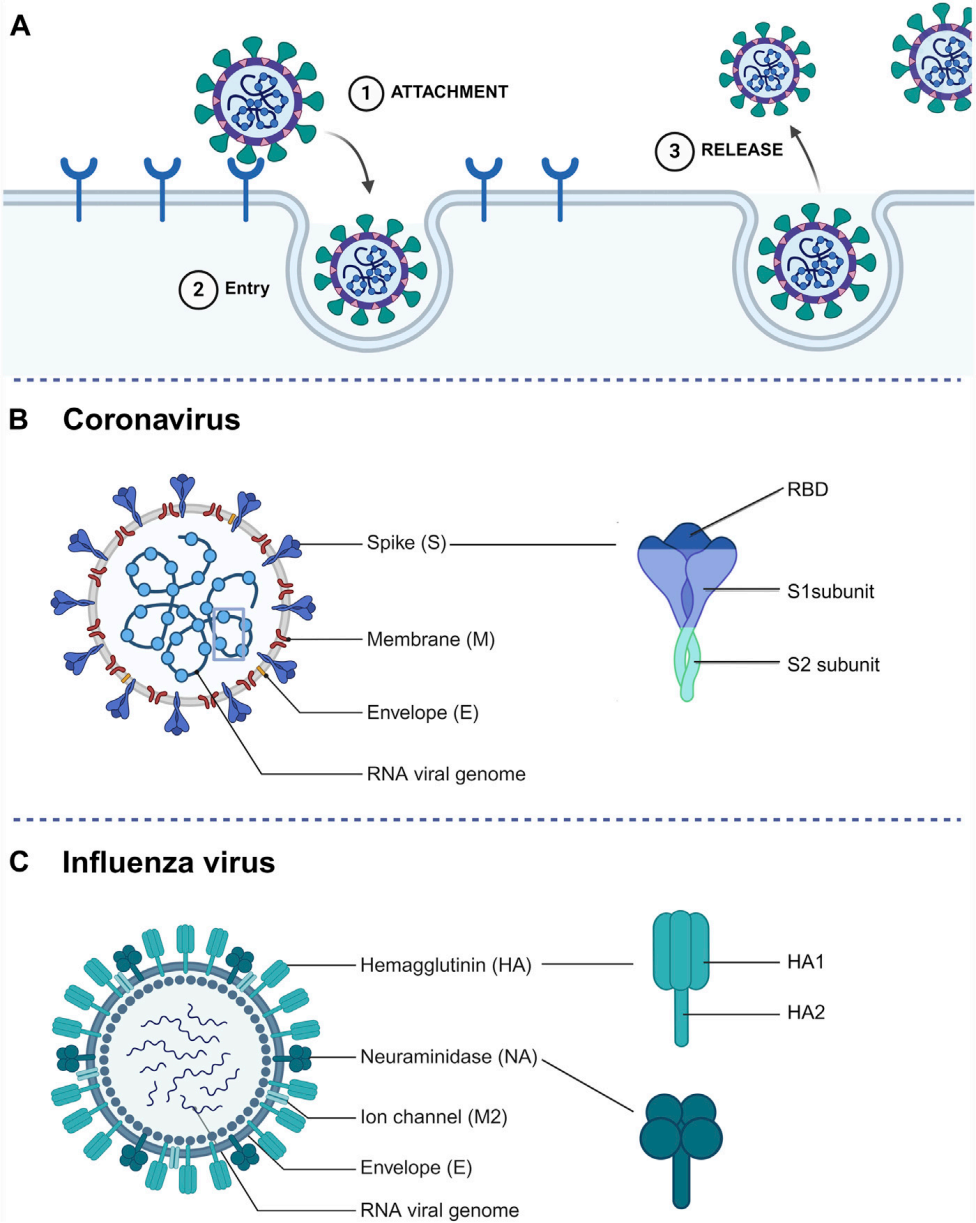
**(A)** Simplified representation of the extracellular steps involved in a standard viral life cycle which can be targeted by sdAbs. **(B)** Schematic representation of a coronavirus particle and spike glycoprotein structure. **(C)** Schematic representation of an influenza A virus particle and surface glycoprotein (hemagglutinin and neuraminidase) structure.

SdAbs have the capacity to disrupt various crucial stages in the viral life cycle, mainly by targeting the viral surface glycoproteins (Figure 2A). To start, the most common interference is to block the virus from interacting with host cell receptors. Additionally, sdAbs can impede virus entry, which in the case of enveloped viruses involves inhibiting fusion with host cells. Lastly, sdAbs can also disrupt the release of newly formed viruses (Wu et al., 2017).

In the subsequent sections, we provide examples of camelid-derived sdAbs targeting CoVs or Influenza viruses. We particularly focus on studies describing innovative optimization approaches and those including *in vivo* data. As multiple reviews on sdAbs targeting SARS-CoV-2 have been published in recent years (Bessalah et al., 2021; Chen et al., 2021; Czajka et al., 2021; Lim et al., 2022; Zebardast et al., 2022; Bhattacharya et al., 2023; Feng and Wang, 2023), our endeavor has not been to give an overview of recent literature, but to integrate the latest findings on optimization techniques described for CoV and Influenza virus targeting sdAbs.

### 3.1 The coronavirus spike protein as sdAb target

The positive-sense RNA genome of CoVs encodes four structural proteins: nucleocapsid (N) protein, membrane (M) protein, envelope (E) protein, and the spike (S) protein (Figure 2B). Among these, the transmembrane S protein emerges as a primary target for CoV-neutralizing sdAbs (Bessalah et al., 2021). The S protein - which folds as a homotrimer - comprises two functional subunits: S1 and S2. It mediates the first and pivotal stage in the viral infection cycle: cell entry. The process of cell entry involves two steps, with the S1 subunit being responsible for the initial step by initiating contact with host cell receptors through its Receptor-Binding Domain (RBD). The RBD serves as a key target for neutralizing sdAbs due to its crucial role in viral entry. SARS-CoV-2, to which the majority of neutralizing sdAbs are targeted, enters the host cell via binding to the angiotensin converting enzyme 2 (ACE2) receptor (Cui et al., 2019; Song et al., 2019). RBD targeting sdAbs can provide robust protection against SARS-CoV-2 in animal models (Huo et al., 2021; Pymm et al., 2021; Aksu et al., 2024). In response to the evolving landscape of SARS-CoV-2, efforts shifted towards selecting sdAbs that target more conserved epitopes on the RBD. By targeting a conserved and cryptic RBD epitope with a hetero-trimeric sdAb, potent SARS-CoV-2 and SARS-CoV neutralization was observed (Hollingsworth et al., 2023). Though the most potent neutralizing sdAbs target the RBD, neutralization can also be achieved by targeting the S1 N-terminal domain (NTD). However, the exact mode of action for NTD neutralizing sdAbs remains undiscovered (Rossotti et al., 2022b; Hollingsworth et al., 2023).

Even though these results seem promising, S1-targeting sdAbs encounter a challenge due to a lack of sequence conservation in this region of the spike proteins, both between and within virus species, thereby limiting their breadth of binding. A promising approach to develop broad-spectrum sdAbs may involve targeting the more conserved S2 subunit. Upon receptor engagement, the S2 subunit facilitates viral entry by mediating fusion of the viral and cellular membranes (Figure 2B). It contains the highly conserved heptad repeat 1 (HR1) and heptad repeat 2 (HR2) domains which form a six-helix bundle during membrane fusion (Walls et al., 2017). S2 targeting sdAbs have shown to potently neutralize multiple SARS-CoV-2 variants *in vitro* and *in vivo* (Rossotti et al., 2022b). Despite a broader binding breadth, S2 targeting sdAbs generally have lower neutralization potency compared to S1 targeting sdAbs (Mast et al., 2021). This observation is complemented by the scarcity of neutralizing epitopes within S2 (Chen et al., 2023; Hollingsworth et al., 2023).

### 3.2 The influenza virus glycoproteins as sdAb target

The segmented negative-sense IAV genome encodes three surface glycoproteins, hemagglutinin (HA), neuraminidase (NA) and matrix-protein 2 (M2) (Figure 2C). IAVs are classified into subtypes based on the (antigenic) properties of their hemagglutinin (HA) and neuraminidase (NA) glycoproteins, and these subtypes are named by their H and N numbers (e.g., H1N1 or H5N1) (Petrova and Russell, 2018). While influenza viruses from different genera can infect humans, IAVs and IBVs cause seasonal epidemics and only IAVs have pandemic potential. The majority of sdAbs described are directed against the glycoproteins of IAV. The trimeric HA protein, containing two subunits HA1 and HA2, is a prevalent target for sdAbs (Figure 2C). HA1, which is the most immunogenic and variable domain of the two, is responsible for host cell receptor binding. SdAbs targeting HA1 have proven to be potent neutralizers of IAV, as demonstrated by a study on a trimerized sdAb capable of potent neutralization of IAV infection (Tillib et al., 2013). The gradual accumulation of mutations on the antigenic sites of HA1 reduces antibody binding and drives antigenic drift. Therefore, targeting the more conserved HA2 region, which facilitates viral membrane fusion, holds promise (Bush et al., 1999; Bommakanti et al., 2010; Jiao et al., 2023). This is confirmed by a study showing that HA2 targeting by an Fc-fused sdAb resulted in full protection of animals against lethal doses of IAVs (Voronina et al., 2022).

A less common target for sdAbs is the homo-tetrameric NA protein which facilitates the release of newly formed virions from infected cells. Although NA-targeting antivirals are typically considered non-neutralizing, they can inhibit the release of virus particles from (decoy) receptors resulting in aggregation of newly assembled virions, thereby delaying virus replication (Palese and Compans, 1976; de Vries et al., 2020). To date, only two studies have described NA-targeting sdAbs. One of them demonstrated potent protection of mice against lethal H5N1 virus infection when administered either prophylactically or therapeutically. In the same study, mice were challenged with an oseltamivir-resistant H5N1 virus, revealing that this sdAb still facilitated complete recovery and ensured the survival of mice. However, it is noteworthy that morbidity was not adequately reduced in this scenario (Cardoso et al., 2014).

The tetrameric transmembrane protein M2, functioning as a viroporin, plays a pivotal role in virus ribonucleoprotein uncoating and release into the host cell cytoplasm (Pinto and Lamb, 2006). A Two-day consecutive treatment using a synthetic M2 targeting sdAb helped reduce, though not fully protect, mice infected with a lethal dose of H3N2 (Wei et al., 2011).

## 4 Optimization strategies for sdAbs

**FIGURE 3:**
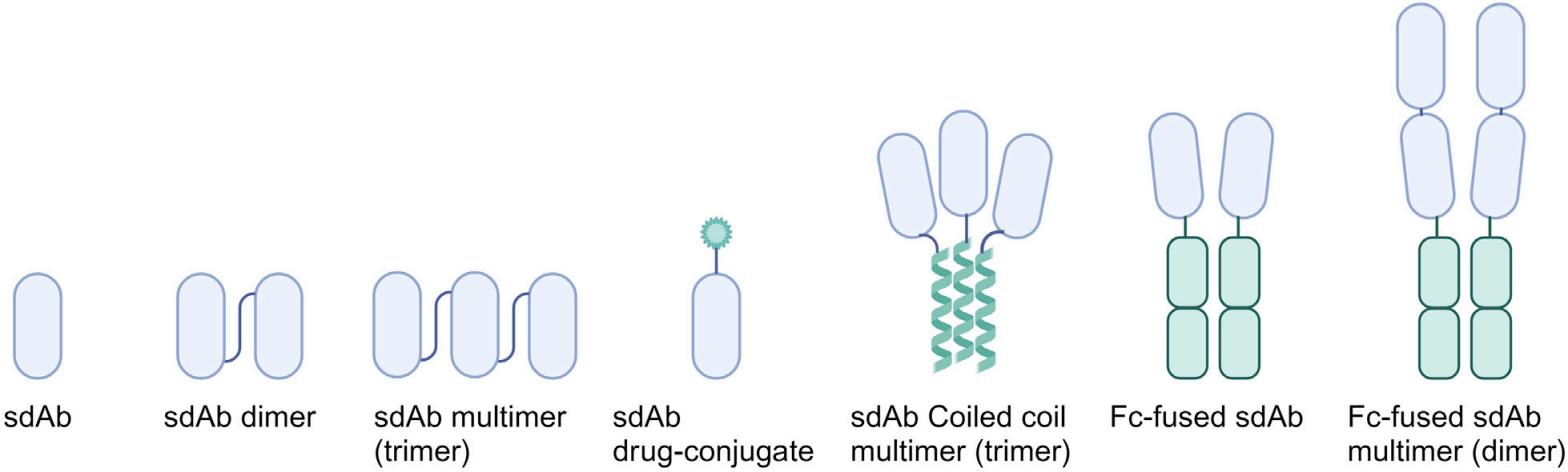
Overview of common sdAb bio-engineering strategies. Multimers are displayed in lowest valency format as example. Genetically linking monovalent sdAbs allows for the generation of sdAb dimers and multimers. By fusing sdAbs to coiled coils, such as GCN4, trimeric sdAbs can be formed. Introduction of immune modulatory effects is achieved by fusing sdAbs to the Fc region. These can be further expanded by fusing sdAb multimers instead of monomers to the Fc region.

SdAbs provide a versatile platform for bioengineering (Figure 3), offering a spectrum of modification methods aimed at enhancing their therapeutic potential against influenza viruses and CoVs (Table 1). Multiple optimization strategies have been explored to increase affinity, enhance neutralization potency and breadth, extend half-life, and introduce immune-modulatory effects to sdAbs.

**TABLE 1:** Overview of optimized sdAbs targeting respiratory zoonotic viruses. Subdivided per optimization approach: multivalency, half-life extension, drug conjugation or immune modulation. Additional information is provided on the target, formatting method, enhancement achieved and *in vitro*/*in vivo* efficacy.

Format, *name*	Virus		Target	Formatting method	Enhancement	*In vitro*	*In vivo*	Reference
Multivalency								
Homo-bivalent *N1-3-VHHb*	IAV		NA	Genetic fusion	Neutralization potency	IC_50_ = 0.157–52.2 nM multiple H5N1 subtypes	Mice, i.n. 60 μg 1 day prior to infection and 6 days post infection: full protection 4LD50 H5N1	Cardoso et al. (2014)
Homo-bivalent *R1a-B6 biv*	IAV		HA	Genetic fusion	Neutralization potency and breadth	IC_50_ = 2.4–36.6 nM H5N1, H1N1, H9N2, H2N2	N.D.	Hufton et al. (2014)
Homo-trimer *aHA-7*	IAV		HA1	Isoleucine zipper domain (ILZ)	Neutralization potency	K_D_ = 0.7 nM H5 IC_50_ = 4.2 nM H5N2	Mice, i.n. 50 μg 2 h prior to, or 24 h post, infection: both show full protection 50LD50 H5N2	Tillib et al. (2013)
Homo-trimer *C5-trimer*	SARS-CoV-2		RBD	Genetic fusion	Neutralization potency	K_D_ = 18 p.m. SARS-CoV-2 RBD IC_50_ = 18 p.m. SARS-CoV-2	Hamster, i.n. 4 mg/kg prior to, or 0.4 mg/kg 24 h post infection: full protection against 104 pfu SARS-CoV-2	Huo et al. (2021)
Homo-trimer *PiN-21*	SARS-CoV-2		RBD	Genetic fusion	Neutralization potency	K_D_ = subpicomolar affinity SARS-CoV-2 RBD IC_50_ = 1.3 p.m. SARS-CoV-2 pseudovirus	Hamster, i.n. 0.2 mg/kg 6 h post infection: full protection 3 × 104 PFU SARS-CoV-2	Xiang et al. (2020), Nambulli et al. (2021)
Homo-trimer *Tribody*	SARS-CoV-2		RBD	COXV-2 human trimerization scaffold	Neutralization potency	K_D_ = subpicomolar affinity SARS-CoV-2 IC_50_ = 2.1 nM SARS-CoV-2 pseudovirus	N.D.	Jiang et al. (2024)
Hetero-trimer *7A9-19B8-S3_29 multimer*	Sarbecovirus		RBD, NTD, S2	Genetic fusion	Neutralization potency	IC_50_ = 1.3–5.7 nM SARS-CoV and SARS-CoV-2 pseudovirus	N.D.	Hollingsworth et al. (2023)
4-armed tetramer *4-arm-PEG Ty1*	SARS-CoV-2		RBD	Strain-promoted azide-alkyne click chemistry (SPAAC)	Neutralization potency	IC_50_ = 13 p.m. SARS-CoV-2 pseudovirus	N.D.	Moliner-Morro et al. (2020)
IgG–VHH Bispecific Antibody *SYZJ001*	SARS-CoV-2		RBD	Genetic fusion	Neutralization potency	K_D_ = 14.8 nM SARS-CoV-2 RBD IC_50_ = 0.026 μg/mL SARS-CoV-2	Mice, i.p. 20 mg/kg 12 h before, or 2 h post infection: full protection 12,000 PFU SARS-CoV-2 mouse-adapted	Chi et al. (2022a)
Half-life extension								
Hetero-dimer with sdAb targeting albumin *Fu2-Alb1*	SARS-CoV-2		RBD and mouse serum albumin	Genetic fusion	*In vivo* protection	N.D.	Mice, i.p. 600 μg 1, 3, 5 and 6 days post infection 86 PFU: full protection SARS-CoV-2	Hanke et al. (2022)
Hetero-trimer with sdAb targeting albumin *Nb15-NbH-Nb15*	SARS-CoV-2		RBD and human serum albumin	Genetic fusion	Half-life and neutralization potency	K_D_ = 0.54 nM (SARS-CoV-2 RBD) and 7.7 nM (HSA) nM IC_50_ = 04 ng/mL SARS-CoV-2 pseudovirus	Mice, i.n. 10 mg/kg 24 h prior, or 1 h post, infection: full protection 1 × 105 PFU SARS-CoV-2. t1/2 = 30 h	Wu et al. (2021)
Ferritin-Displayed sdAb *Fenobody*	IAV		N.D.	Genetic replacement of ferritin helix ε + loop by sdAb	Half-life	K_D_= 0.243 HAU/mL H5N1	Mice, intravenous 2 nmols at selected time points. t1/2 = 326.3 min	Fan et al. (2018)
Drug conjugation								
SdAb Zanamivir conjugate *VHHkappa-zanamivir*	IAV and IBV		NA and mouse kappa light chain	Sortase-mediated	Half-life (zanamivir) and *in vivo* protection	K_D_ = 6–13 nM (NA)	Mice, i.p. 1 mg/kg 1 h prior to infection: full protection 10LD50 H1N1. t1/2 = 84.1 h	Liu et al. (2023b)
SdAb coupled to cGAMP containing liposomes *VHH-Lip/cGAMP*	SARS-CoV-2		RBD and STING	Direct coupling of thioether sdAbs to cGAMP containing liposomes	N.D.	IC_50_ = 0.76 µM SARS-CoV-2 pseudovirus	N.D.	Zhou et al. (2023)
Immune Modulation								
Monomer fused to Fc *WNbFc 36*	SARS-CoV-2		RBD	Genetic fusion	N.D.	IC_50_ = 0.1 nM SARS-CoV-2	Mice, i.p. 5 mg/kg 1 day prior to infection: full protection SARS-CoV-2	Pymm et al. (2021)
Monomer fused to Fc *G2.3-Fc*	IAV		HA2	Genetic fusion	Immune effector function and neutralization potency and breadth	IC_50_ = 1.8 nM H1N1	Mice, i.n. 0.6 mg/kg 1 h prior to, or 2 mg/kg 2 h post, infection: full protection 5LD50 H1N1 and H5N2	Voronina et al. (2022)
Monomer fused to Fc *Mred05*	SARS-CoV-2		RBD	Genetic fusion	Neutralization potency	K_D_ = 0.62 nM SARS-CoV-2 IC_50_ = 0.17 nM SARS-CoV-2	Hamsters i.p. 1 mg 1 day prior to infection: full protection SARS-CoV-2	Rossotti et al. (2022b)
Monomer fused to Fc *huR3DC23-Fc_LS*	SARS-CoV-2		S2	Genetic fusion	Immune effector function and neutralization potency	IC_50_ = 2.5 ng/mL SARS-CoV-2	Mice, i.p. 2 mg/kg 4 h post infection: strong restriction of 100 TCID50 SARS-CoV-2 replication	(De Cae et al., 2023) *Preprint*
Hetero-dimer fused to Fc *Nb-X2-Fc*	SARS-CoV-2		RBD	Genetic fusion	Neutralization potency and breadth	IC_50_ = 1.8 nM SARS-CoV-2	N.D.	Yang et al. (2023)
Hetero-tetramer fused to Fc *MD3606*	IAV and IBV		HA	Genetic fusion	Immune effector function and neutralization potency and breadth	K_D_= <0.1–3.8 nM multiple HA subtypes IC_50_ = 1–40 nM multiple IAVs and IBVs	Mice, intravenous 5 mg/kg 1 day prior to infection: full protection 25LD50 H1N1, H3N2, H7N9, IBV	Laursen et al. (2018)
Hetero-trimer fused to Fc *ABS-VIR-001*	SARS-CoV-2		RBD	Genetic fusion	Neutralization potency	K_D_ = 0.095 nM SARS-CoV-2 S1 IC_50_ = 6.44 nM SARS-CoV-2 pseudovirus	Mice, i.p. 10 mg/kg 2 h post infection: full protection 1000 PFU SARS-CoV-2. I.n. 25 mg/kg 10 h prior to infection 75% protection 1000 PFU SARS-CoV-2	Dong et al. (2020), Titong et al. (2022)
Homo-decamer fused to IgM Fc *MR14*	SARS-CoV-2		RBD	Genetic fusion	Neutralization potency and breadth and half-life *in vivo*	IC_50_ = 91 ng/mL SARS-CoV-2	Mice, i.n., 5 mg/kg 6 h prior to, or 6, 30, and 54 h after, infection: effective protection against SARS-CoV-2 BA.2. t1/2 = 28.1 h	Liu et al. (2023a)
Homo-hexamer fused to Fc *hexavalent VHH-72*	SARS-CoV-2		RBD	Genetic fusion	Neutralization potency	IC_50_ = 0.035 nM SARS-CoV-2 pseudovirus	N.D.	Zupancic et al. (2021)

The most prevalent optimization strategy involves leveraging sdAb avidity effects through multimerization, often achieved by genetic fusion. For example, compared to its monomeric counterpart, a genetically-fused trimeric sdAb demonstrated a remarkable 30-fold improvement in neutralization efficacy against SARS-CoV-2 (Xiang et al., 2020). A follow-up study revealed that this homo-trimeric sdAb was not only highly effective *in vitro* but also demonstrated effective control of infection *in vivo* in a hamster model through intranasal administration (Nambulli et al., 2021). Using a different conjugation method, namely, click chemistry, a SARS-CoV-2 targeting sdAb was conjugated to 4-arm PEG scaffolds. The resulting PEG-based tetrameric construct showed over a 1000-fold increase in neutralization potency against live SARS-CoV-2 virus (Moliner-Morro et al., 2020). In addition to enhancing potency, sdAb multimerization can lead to an increase in breadth. As has been observed in a study involving an HA-targeting sdAb selected against H1N1, which, in homo-bivalent format, gained the ability to neutralize H2N2 (Hufton et al., 2014). Similarly, a hetero-tetrameric sdAb demonstrated an increase in neutralization breadth against multiple IAVs and IBVs compared to its monovalent sdAb components (Laursen et al., 2018).

Because of their small size, monovalent sdAbs exhibit rapid renal clearance and short half-lives typically ranging from 1 to 3 h (Cortez-Retamozo et al., 2002). As frequent administration is undesirable, and in alternative to constructing multimers, various strategies have been explored to extend their duration of action. Many of these approaches rely on linking the sdAb to serum albumin, which has a serum half-life of 3 weeks and therefore can significantly extend the half-life of small-sized drugs (Sleep et al., 2013). While direct conjugation of serum albumin to sdAbs is a straightforward option, it presents challenges as it significantly increases overall particle size with 66.5 kDa, potentially impacting inhibitory effects or complicating administration. To circumvent this issue, an alternative method employs fusing the sdAb to an albumin-targeting sdAb, rendering the interaction with albumin reversible. This approach has demonstrated effectiveness in a study where a serum albumin-binding sdAb was fused to a virus-specific sdAb. The resulting bispecific construct showed potent therapeutic *in vivo* protection, potentially due to an extended half-life (Hanke et al., 2022). This approach is equally applicable to higher valency sdAb constructs, as highlighted by a study on a trimeric sdAb construct where inclusion of an albumin-targeting sdAb resulted not only in extended half-life, but also increased sdAb concentrations in relevant tissues and enhanced *in vivo* protection (Wu et al., 2021).

Extending sdAb serum half-life is also achievable through the addition of an Fc-domain, due to the increase in size and through the interaction with the neonatal Fc receptor (FcRn) present on endothelial cells, which protects IgG from lysosomal degradation (Kontermann, 2009). Moreover, addition of an Fc-domain automatically dimerizes sdAbs thereby increasing avidity and potentially neutralization potency. Another rationale for introducing an Fc-tail lies in its capacity to confer immune modulatory effects. Adding the Fc region can result in the initiation of Fc-mediated effector functions due to interaction with either Fcγ receptor (FcγR) on the surface of immune cells or the complement component 1q (C1q) protein. FcγR interaction can initiate antibody-dependent cellular cytotoxicity (ADCC) or antibody-dependent cell-mediated phagocytosis (ADCP), whilst interaction with C1q leads to complement-dependent cytotoxicity (CDC) (Nimmerjahn and Ravetch, 2008; Abdeldaim and Schindowski, 2023). It has been shown that sdAb Fc-domain addition can significantly improve *in vivo* protection against IAV and IBV, explained by the initiation of ADCC mediated protection due to the strong activation of FcγRIIIa (Laursen et al., 2018). In some cases the addition of an Fc-domain can even add neutralization potency to previously non-neutralizing sdAbs, as exemplified by a monomeric SARS-CoV-2 targeting sdAb (Rossotti et al., 2022b).

Another optimization strategy involves the conjugation of antiviral drugs to sdAbs, enhancing their overall effectiveness. While sdAb drug conjugates have been extensively investigated in the cancer field, this approach has been less explored for sdAbs targeting respiratory zoonotic RNA viruses. Examples include linking Zanamivir, a neuraminidase inhibitor, to a sdAb that recognized the kappa light chains of mouse immunoglobulins, facilitating the recruitment of polyclonal Igs to NA-expressing, infected cells. This bispecific construct extended therapeutic benefits in mice and enhanced ADCC and CDC, and effectively protected mice against IAV and IBV strains (Liu et al., 2023b). In another study, liposomes carrying cGAMP, a stimulator of interferon genes (STING) antagonist, were linked to SARS-CoV-2 targeting sdAbs, to direct the liposomes to the site of infection. While this complex only showed a 5x increase in pseudovirus neutralization compared to sdAb alone, it might still show a benefit in pre-clinical and clinical settings, as the novelty of the approach lies in overcoming delivery limitations of cGAMP to the cytoplasm by using sdAb targeted liposome delivery (Zhou et al., 2023).

## 5 Challenges and considerations in sdAb usage

Both CoVs and influenza viruses mainly affect the respiratory tract in humans. SARS-CoV-2 causes both lower and upper respiratory tract infections and influenza viruses mainly lead to upper respiratory tract infections, which can extend to the lower airways in more severe cases (Short et al., 2014; Hughes et al., 2023). For treating airway infections, systemic and intranasal administration are the main routes. Intranasal delivery is preferred for localized high concentrations, faster onset, and reduced systemic exposure, while systemic administration requires higher dosages and can be less efficient for airway drug delivery (Fortuna et al., 2014). Due to its small size, high solubility, thermal resistance and overall robustness, sdAbs can be nebulized into inhalable aerosols for efficient intranasal administration (Van Heeke et al., 2017).

One common concern in sdAb optimization is that it usually leads to an increase in size, potentially affecting suitability for intranasal administration. However, various studies demonstrate that the efficiency of intranasal administration extends to higher valency constructs (Tillib et al., 2013; Huo et al., 2021; Nambulli et al., 2021; Wu et al., 2021). Additionally, an Fc-fused sdAb has been succesfully aerosolized (Voronina et al., 2022), and even a large decameric sdAb construct could be nebulized resulting in effective *in vivo* protection (Liu et al., 2023a). These observations align with an even larger variety of studies showcasing successful protection against respiratory CoVs and influenza viruses through intranasal sdAb administration (table 1) (Tillib et al., 2013; Cardoso et al., 2014; Huo et al., 2021; Nambulli et al., 2021; Wu et al., 2021; Voronina et al., 2022; Liu et al., 2023a). Some studies even emphasize additional benefits compared to intraperitoneal or intravenous administration. For instance, intranasal administration of sdAbs can lead to faster recovery of infected animals (Huo et al., 2021). Additionally, intranasal administration can lead to higher bio-availability of sdAbs in the respiratory tract, a tissue which is in some cases only reached after intranasal administration (Wu et al., 2021). Intranasal delivery can also result in pathological benefits as exemplified by a study where they found alleviated lung lesions after intranasal sdAb administration (Liu et al., 2023a). In some cases both intranasal and intraperitoneal sdAb administration results in full *in vivo* protection, where the advantage of intranasal administration is the requirement of a lower dose (Voronina et al., 2022).

As this review underlines, Fc-domain addition is a frequently used sdAb formatting strategy. A drawback of the Fc-tail addition is the increase in sdAb size and also the loss of ability to produce them in low-cost bacterial system. An alternative would be to conjugate viral targeting sdAbs to FcγRIII-targeting sdAbs. Similar to Fc-appended sdAbs, such a bispecific construct would still allow recruitment of immune cells including natural killer cells, whilst retaining its small size and producibility in eukaryotic cells. This method has already been explored in the cancer field, where bi-specific constructs consisting of a cancer targeting and a FcγRIII-targeting sdAb elicited potent ADCC responses (van Faassen et al., 2021; Nikkhoi et al., 2022).

A challenge in clinical sdAb usage is the potential development of anti-drug antibodies (ADAs) after repeated sdAb administration, which could compromise treatment efficacy and lead to adverse effects. Until now, ADA formation in the context of antiviral sdAbs has not been extensively studied. Nevertheless, broader sdAb research indicates limited immunogenicity of humanized sdAbs, as demonstrated by preclinical mice studies on TNFa targeting sdAbs and clinical trials on an sdAb targeting respiratory syncytial virus (Bruyn et al., 2015; Ishiwatari-Ogata et al., 2022). Conversely, in another clinical I trial, patients receiving a humanized tetravalent sdAb targeting death receptor five experienced severe liver damage, leading to the early termination of the trial. It remains unclear whether pre-existing and emerging immunogenicity played a role in the hepatoxicity observed (Papadopoulos et al., 2015). Overall, in both pre-clinical and clinical studies, involving primarily humanized sdAbs, findings consistently indicate limited ADA formation, with neutralizing ADAs being scarce (Rossotti et al., 2022a).

Furthermore, repeated sdAb administration may exert selective pressure, potentially leading to viral escape. To mitigate this challenge, preparation of sdAb cocktails has been proposed; however, studies present contrasting findings (Koenig et al., 2021; Mast et al., 2021). Multivalent sdAb constructs targeting diverse epitopes show promise in mitigating viral escape. A recent study demonstrated that only biparatopic sdAbs, targeting two non-overlapping epitopes on the same target, efficiently hamper the emergence of SARS-CoV-2 pseudoviral escape mutants, in contrast to the monovalent sdAbs, homo-multivalent sdAbs, or sdAb cocktails used in the study (Koenig et al., 2021). A synthetic biparatopic sdAb demonstrated a similar effect, with resistant viruses emerging rapidly in the presence of the single monovalent sdAbs, while no escape variants were observed in the presence of the biparatopic sdAb (Walter et al., 2022). Both studies highlight the intrinsic advantage of biparatopic molecules, as the barrier for resistance is inherently higher due to the necessity of the virus acquiring mutations in two epitopes simultaneously.

## 6 Conclusion and future perspectives

Since the discovery of heavy chain antibodies in 1993, the rapid development of sdAbs has culminated in the market authorization of Caplacizumab, a von Willebrand factor inhibitor, by the EMA and FDA in 2018 and 2019 respectively (Duggan, 2018). SdAbs offer unique advantages such as their small size, cost-effective production, robustness, and ease of bioengineering. As highlighted by this review, sdAbs have been optimized using various strategies leading to enhanced neutralization potency and breadth, half-life, and immune modulation.

In animal models, the intranasal delivery of (optimized) sdAbs for the treatment of respiratory zoonotic viruses has shown promise, necessitating lower doses compared to intraperitoneal and parental administration, displaying heightened bioavailability in relevant tissues and mitigating adverse pathological changes (Huo et al., 2021; Wu et al., 2021; Voronina et al., 2022). The prophylactic and therapeutic inhalation of sdAbs in high-risk individuals could be a valuable strategy during upcoming endemics and pandemics. Moreover, promising sdAbs with increased breadth have been achieved by engineering of multiparatopic sdAbs (Koenig et al., 2021; Walter et al., 2022). Additionally, targeting conserved parts on viral glycoproteins provides the potential to combat future-emerging variants or related viruses. Optimization strategies can be implemented to improve or add neutralization potential to these sdAbs targeting conserved domains (Rossotti et al., 2022b).

Despite demonstrating potency in *in vitro* and preclinical studies, the potential of sdAbs to prevent virus infection and disease in clinical settings remains to be determined. Only an sdAb targeting respiratory syncytial virus has entered a clinical phase I trial, and there is currently no approved virus-targeting sdAb for clinical use (Bruyn et al., 2015). The recent COVID-19 pandemic not only highlights the pandemic threat posed by zoonotic RNA viruses, but has also provided valuable insights into the primary response to newly emerging human infecting viruses. As our review emphasizes, sdAbs offer several advantages over traditional antibodies. In case of a pandemic, requiring rapid worldwide treatment, sdAbs present a big advantage due to their cost-efficient and rapid production compared to the costly, time-consuming and labor intensive process of traditional antibody production (Chames et al., 2009; Liu and Huang, 2018). Additionally, their high stability allows for storage at room temperature, easing both storage and worldwide distribution (Kunz et al., 2018). Still, during the course of the pandemic only antibodies were approved, with Emergency Use Authorizations granted (Hwang et al., 2022). To date, no SARS-CoV-2 targeting sdAbs reached the clinic. There is no evident explanation for this discrepancy, except for the fact that antibodies have a more established profile. Antibodies, initially discovered as protective substances in the 1880s and named in 1891, received their first FDA and EMA approval in 1986 and 1991 respectively (Van Wauwe et al., 1980; Rosenberg, 1994; Marks, 2012). Considering this historical context, it is not surprising that sdAbs, as relatively new antigen-targeting moieties, are not yet widely used. Nevertheless, it is encouraging to see a lot of efforts were made towards identifying potent sdAbs targeting SARS-CoV-2, alongside the fast discovery of antibodies during the pandemic. However, there remains an urgent need for further research to understand the overall efficacy of sdAbs in clinical settings and the effects of optimization strategies on clinical outcomes. Areas requiring additional investigation include the long-term effects of repeated sdAb administration and the immunogenicity of sdAbs in clinical applications. The growing interest and increasing number of clinical trials expected to be conducted will likely facilitate the clinical translation of sdAbs. Hopefully this will enable sdAbs to emerge as first-line antiviral treatments in the likely event of future pandemics, either independently or alongside of traditional antibodies.

In conclusion, this review discusses the recent advances in the field of sdAb bioengineering in the fight against respiratory zoonotic viruses. Their unique attributes such as compact size, cost-effective production, versatile bioengineering capabilities and potential for intranasal delivery has shown promise in pre-clinical research for developing more effective and targeted therapeutic interventions during respiratory virus outbreaks. Overall, the rapid developments in sdAbs optimization holds the potential to outpace escape mechanisms of respiratory zoonotic viruses, offering an exciting avenue for future research and application in the field of antiviral therapeutics.
